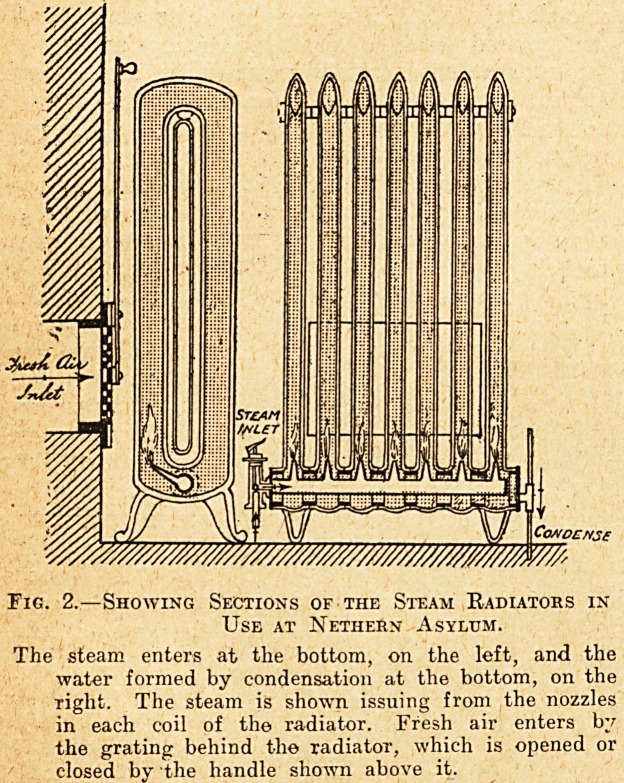# The Nethern Asylum

**Published:** 1917-05-19

**Authors:** 


### THE HEATING OF HOSPITALS.

### XII. The Nethern Asylum.

### VERY LOW PRESSURE STEAM WITHOUT VACUUM PUMPS; THE PLENUM SYSTEM; SMALL INDEPENDENT STEAM BOILERS; AND THE FURNACE HOT-AIR SYSTEM.

The Nethern Asylum, situated near Coulsdon in Surrey, uses all the systems mentioned above. The principal feature in the heating system is the very low pressure steam, at $1\frac{1}{2}$ lb. per square inch, which is used in a special form of radiator, designed to produce the same temperature as the hot-water system. It will be remembered that several hospitals are heated by low-pressure steam in radiators at about 5 lb. per square inch; the pressure inside the radiators being lowered by producing a partial vacuum in the system of pipes leading back to the vessel from which the boiler supply is taken. Lowering the pressure of the steam lowers its temperature, so that the temperature of the surfaces of the radiators is at about the same figure, 160° to 180° F., as with the hot-water system.

At the Nethern Asylum there are two large blocks, for males and females respectively, which are heated by the steam radiators described below; the acute block, the idiots' block, the recreation hall, and the chapel have their own separate systems of heating. The building is lit by electric light, and the exhaust steam from the lighting engines is used, as far as it will go, for heating the male patients' block.

Fig. 1.—Section of Air-heating Apparatus used for Warming the Chapel at Nethern Asylum.

The furnace is self-feeding, within the capacity of the coal-hoppers shown. *A*, Combustion Chamber; *B*, Distributing Pipe; *C*, Heating Elements; *D*, Smoke Boxes; *K*, Cold-air Channels; *P.P.*, Cleaning Doors; *S*, Flue to Chimney; *W*, Warm-air Channels; *V*, Water Pan; *F*, Water Cock; $T_1$, $T_2$, $T_3$ Hopper Doors.

#### The Boilers.

There are three Lancashire boilers, each 32 feet long by $7\frac{1}{2}$ feet in diameter, working up to a pressure of 120 lb. per square inch. Two of the boilers are sufficient to meet all requirements, the third is usually kept in reserve. The steam from the boilers is taken to the hot-well room, under the floor of the boiler-house, where reducing apparatus is fixed, which lowers the pressure of the steam to 3 lb. per square inch. The exhaust steam from the electric-light engines is brought to the same point, and passed through similar reducing apparatus. There is an oil separator provided for the exhaust steam, to remove the oil from the steam before it enters the heating system. The steam at 3 lb. per square inch is carried in pipes, fixed in subways on roller brackets and provided with expansion joints, to the male and female blocks. In the basement of the two blocks the steam is passed through another reducing apparatus, which lowers its pressure to $1\frac{1}{2}$ lb. per square inch; at this pressure it is carried by the usual pipe system to the radiators, which are shown in section in fig. 2. In outward appearance they are similar to hot-water and other steam radiators; the special feature in connection with them is the steam nozzle, as it is called, fixed in each coil of the radiator. Nozzles are designed specially to create an expansion of the steam as it issues from them; in addition, it is claimed that the steam issuing from the nozzles causes a circulation of steam and air through each coil of the radiator, resulting in a lowering of the temperature to the figure named above. The steam enters at one side of the radiator at the bottom, as shown in the figure, and the water formed from the condensed steam leaves the radiator by a pipe on the other side at the bottom, and finds its way by gravity to the hot well under the boiler-house; thence it is delivered by the usual boiler feed pump into the boiler. The condensed water from the female block cannot be carried back by gravity, and is therefore pumped back to the hot well by two automatic pumps.

The dormitories, day-rooms, etc., are heated by radiators fixed in the usual positions, with gratings opening to the atmosphere behind them, as shown in fig. 2; the gratings are arranged to open or shut by hand. All the radiators in the patients' rooms are protected by galvanised wirework on galvanised iron ribs, fixed to the floors and wall, but arranged to be easily removed. Ventilation is by means of

Fig. 2.—Showing Sections of the Steam Radiators in Use at Nethern Asylum.

The steam enters at the bottom, on the left, and the water formed by condensation at the bottom, on the right. The steam is shown issuing from the nozzles in each coil of the radiator. Fresh air enters by the grating behind the radiator, which is opened or closed by the handle shown above it.

the fresh air entering behind the radiators and leaving by the flues of the open fireplaces; in some cases separate grates for the extraction of the foul air are fitted near the floor line. Seven hundred and fifty cubic feet of air is provided per patient in the ordinary rooms and dormitories; 1,000 cubic feet is provided in the sick and infirm ward. The administrative and entrance blocks are warmed partly by radiators and partly by warm air from the subways.

### The Recreation Hall.

The recreation hall is warmed and ventilated on the Plenum system. A battery of gilled pipes, through which hot gases from a furnace are passing, is placed in a chamber under the hall, and fresh air is passed over the battery by an electrically driven fan. The warm air enters the hall by ports provided for it, about 8 feet above floor level; the vitiated air is extracted through grates and ducts near the floor line, and carried off by separate flues. The air of the hall is changed twice an hour in very cold weather, and three times an hour in mild weather.

### The Acute Hospital and the Idiots' Block.

These two buildings are heated by radiators similar to those in the large blocks, but supplied with steam by special boilers having hopper, self-feeding furnaces and automatic draught regulators, so that they require very little attention. The steam is generated at from 1½ to 2 lb. per square inch, and is taken direct to the radiators by the usual pipe system.

### The Chapel.

The chapel is heated by hot air, the apparatus employed being what the makers call a calorifier, though it is quite different from the steam calorifiers that have been described in these articles. One of them is shown in fig. 1. As will be seen, it is the hot gases from the furnace which provide the heat, the air to be heated being passed over the pipes through which the hot gases flow.

## Figures and Tables

**Fig. 1. f1:**
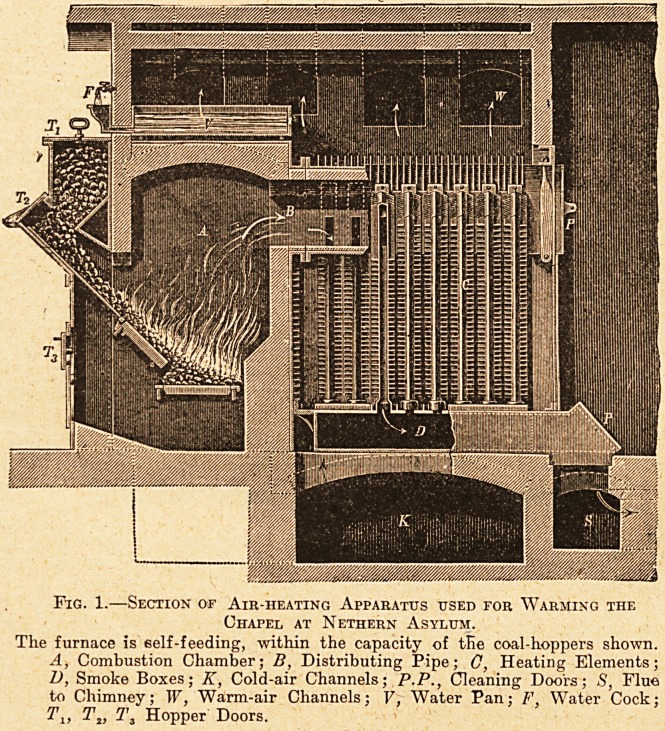


**Fig. 2. f2:**